# Telehealth insulin titration in adults with diabetes: a randomized controlled trial comparing bluetooth-enabled versus traditional glucometers

**DOI:** 10.3389/fendo.2025.1724811

**Published:** 2025-12-16

**Authors:** Xia Lian, Hui Ling Liew, Ying Shan Lee, Anita Ying Lin, Eunice Yu Wen Goh, Christina Yang Hoon Goh, Hwee Chen Quek, Isaac Jun Song Tan, Helen Lim, Ying Jie Chee, Zhi Han Quek, Liang Shen, Rinkoo Dalan

**Affiliations:** 1 Department of Nursing, Tan Tock Seng Hospital, Singapore, Singapore; 2 Alice Lee Centre for Nursing Studies, Yong Loo Lin School of Medicine, National University of Singapore, Singapore, Singapore; 3 Department of Endocrinology, Tan Tock Seng Hospital, Singapore, Singapore; 4 Biostatistics Unit, Yong Loo Lin School of Medicine, National University of Singapore, Singapore, Singapore; 5 Lee Kong Chian, School of Medicine, Nanyang Technological University Singapore, Singapore, Singapore

**Keywords:** telehealth, blood glucose self-monitoring, glycemic control, diabetes mellitus, bluetooth

## Abstract

**Objective:**

Blood glucose self-monitoring is crucial for individuals with diabetes mellitus and on insulin therapy to ensure safe glycemic control and optimal treatment outcomes. This study evaluates the effectiveness of Bluetooth-enabled glucometers (BTG) versus Traditional glucometers (TG) in a telehealth insulin titration program for individuals with diabetes.

**Methods:**

This 24-week, open-label, randomized controlled trial enrolled 120 participants with diabetes from a tertiary hospital. Participants, aged 21–70 years, who required either insulin initiation or intensification were randomly assigned to either the BTG or TG group. Both groups received three biweekly teleconsultations with Diabetes Nurse Educators for insulin dose adjustments, followed by two clinic visits at three-month intervals.

**Results:**

Participants were predominantly male, Chinese, and diagnosed with Type 2 diabetes. Both groups demonstrated significant reductions in glycated hemoglobin (HbA1c) throughout the study. The TG group achieved HbA1c reductions of 2.8% at Week 12 and 3.1% at Week 24 (both *p* < 0.001), while the BTG group showed reductions of 2.23% and 2.18% respectively (both *p* < 0.001). There were no significant between-group differences in HbA1c at any time point. However, the BTG group showed significantly fewer emergency department visits than TG (4.1% vs. 16.7%, *p* = 0.039). Both groups demonstrated improvements in diabetes-related distress, with no significant differences between groups.

**Conclusion:**

BTG did not demonstrate glycemic superiority over TG in telehealth insulin titration; however, its association with reduced emergency department visits suggests potential benefits for healthcare utilization. Future studies should investigate the integration of BTG with comprehensive diabetes care platforms, with a focus on long-term outcomes and cost-effectiveness.

**Clinical Trial Registration:**

https://www.isrctn.com/ISRCTN69173566, Identifier: ISRCTN69173566.

## 1 Introduction

Diabetes Mellitus is a major global health challenge, affecting over 537 million people worldwide in 2021, with projections indicating an increase to 783 million by 2045 (1). Poor glycemic control significantly increases the risk of long-term complications, including cardiovascular disease, diabetic retinopathy, nephropathy, and neuropathy (2). Insulin therapy is crucial for individuals who fail to achieve glycemic control despite maximum oral hypoglycemic agents. For those newly started on insulin therapy or undergoing insulin intensification, close glucose monitoring during the initial weeks is crucial to prevent adverse glycemic events and enable timely treatment adjustments (3, 4).

In Singapore, Diabetes Nurse Educators (DNEs) provide structured education to individuals with diabetes on self-monitoring of blood glucose (SMBG). The traditional glucometer requires individuals to perform finger-prick testing and manually record their readings in physical logbooks. During clinic visits, patients present their glucose logbooks to DNEs for review, who analyze glucose trends and formulate management plans. However, this process is often burdensome, time-consuming, and prone to errors, which can result in inaccurate data recording and delays in clinical decision-making (5).

The COVID-19 pandemic has accelerated the widespread adoption of telehealth services, aiming to reduce face-to-face clinic visits while maintaining equivalent clinical outcomes (6). However, traditional telehealth consultations present significant challenges for diabetes care. During virtual sessions, DNEs must obtain each glucose reading verbally from patients and manually transcribe them into medical records. This process is both time-consuming and prone to transcription errors (7). This challenge is particularly pronounced when managing elderly individuals with hearing impairments, where communication barriers can further compromise data accuracy.

Bluetooth-enabled glucometers (BTG) address these limitations by wirelessly transmitting blood glucose readings to smartphone applications, eliminating the need for manual recording and reducing the risk of human error (8). BTG enables remote monitoring, allowing healthcare providers to track individuals’ glucose levels and intervene when necessary. Instead of spending valuable time collecting and recording readings, DNEs can focus on meaningful discussions about glucose patterns, lifestyle modifications, and insulin adjustments (9, 10). Trained DNEs follow established algorithms and protocols for insulin titration (**Supplementary Data Sheet 1**, **2**).

While Continuous Glucose Monitoring (CGM) systems have become widely adopted internationally over the past decade, with insurance coverage making sensors financially accessible in many countries, the situation in Singapore differs. Here, patients must bear the full cost of CGM sensors out of pocket, which has limited their widespread adoption (11). Consequently, traditional finger-prick blood glucose testing remains the predominant approach for glucose monitoring.

Studies have demonstrated that Telehealth Insulin Titration Programmes (TITP) significantly improve glycemic control through safe and effective insulin adjustments by trained nurses over the telephone (12, 13). Beyond addressing clinical inertia, these programmes reduce emotional distress associated with diabetes management by empowering patients with knowledge, strengthening their self-management skills, and minimising the need for frequent clinic visits.

This randomized controlled trial evaluates whether Bluetooth-enabled glucometers (BTG) improve glycemic control compared to traditional glucometers (TG) within a Telehealth Insulin Titration Program (TITP). The study also compares their impact on cardiometabolic outcomes, and diabetes-related distress. We hypothesise that BTG integration within structured telehealth support will enhance glycemic control, reduce diabetes-related emotional distress, and improve the efficiency of remote insulin titration processes, ultimately optimizing patient care delivery and healthcare resource utilisation. These findings will provide valuable insights for optimising telehealth diabetes management, with implications for both patient care and healthcare resource utilisation.

## 2 Methodology

### 2.1 Study design

We conducted a 24-week, open-label, randomized controlled trial with two parallel arms at a tertiary hospital in Singapore. The study consisted of an initial 6-week telehealth intervention period, followed by an 18-week follow-up phase. The trial was registered on the ISRCTN Registry (ISRCTN69173566).

### 2.2 Study population

Participants were recruited during inpatient admissions, after review by the hospital’s Integrated Diabetes Care Programme team, or outpatient clinic visits. Eligible participants were aged 21–70 years and met one of three criteria: they were newly initiated on insulin, undergoing insulin regimen intensification, or had existing insulin dose changes exceeding 20%. All participants were required to own a smartphone and be familiar with mobile applications. The study excluded individuals who were pregnant, had cognitive impairment, were unable to operate a glucometer, were unwilling to perform blood glucose monitoring or participate in teleconsultation, or were already enrolled in other diabetes-related research involving glucose monitoring and medication titration. Informed consent was obtained from all participants.

### 2.3 Sample size

The sample size was calculated to detect a difference in the change from baseline glycated hemoglobin (HbA1c) between the intervention and control groups. Based on the study by Hompesch et al. (14), which utilized the MySugr^®^ mobile application (Roche Diabetes Care GmbH, Mannheim, Germany) in Type 1 diabetes, with a difference in mean blood glucose levels of 37.7 mg/dL (corresponding to an estimated HbA1c of 1.3%), assuming the standard deviation (SD) of HbA1c was about 2.25%. Considering a 10% dropout rate, the target sample size was 120, with 60 participants in each group.

### 2.4 Randomization

The randomization list was pre-generated by an independent statistician using a computer-generated sequence to ensure unbiased group assignment. Eligible participants were randomly allocated in a 1:1 ratio to either the Bluetooth-enabled glucometer (BTG) group or the traditional glucometer (TG) group. Allocation concealment was ensured through an automated computerized system, in which group assignment was revealed only after the study team entered each participant’s enrolment details. This process prevented foreknowledge of allocation and minimized selection bias. Due to the nature of the intervention, neither the study team members nor the participants were blinded to group assignment.

### 2.5 Interventions

**Figure 1:**
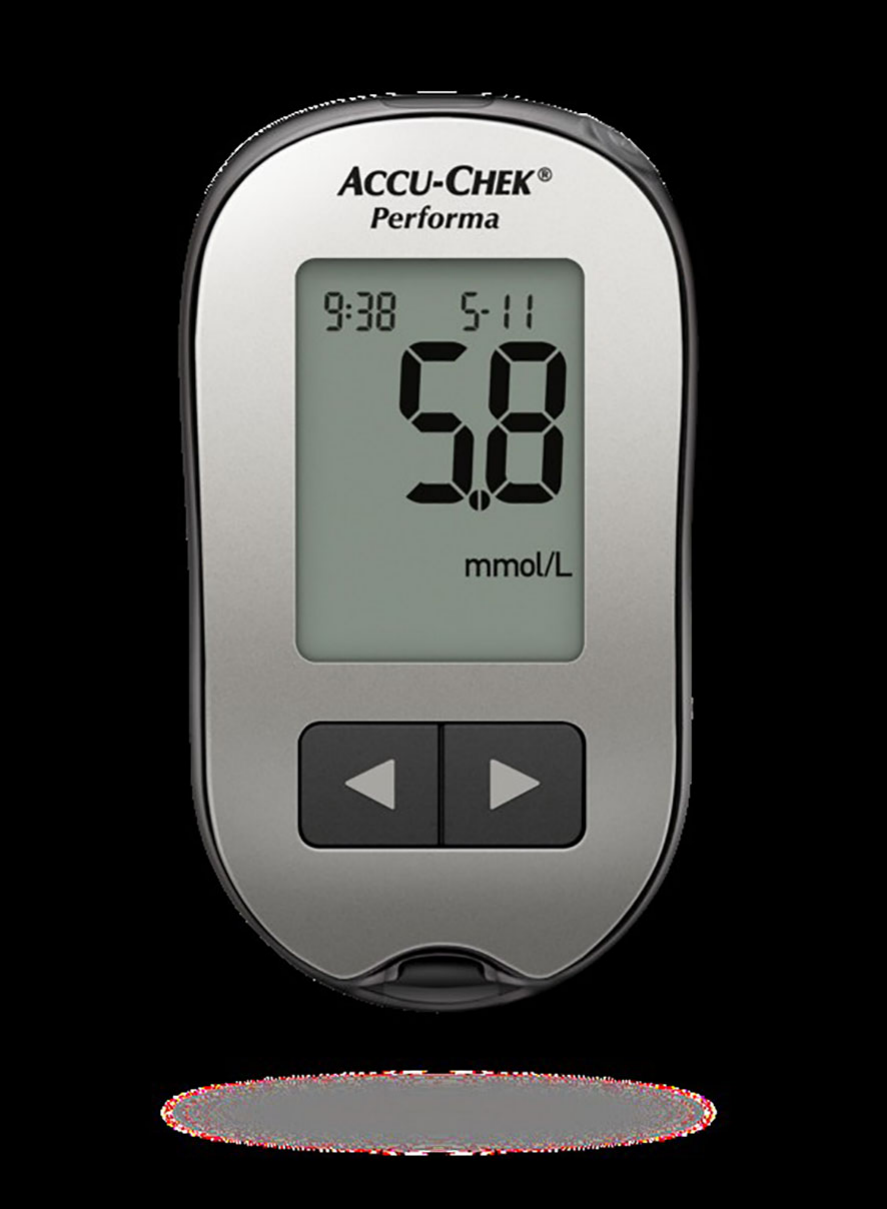
The control group used the Accu-Chek^®^ Performa glucometer (Roche Diabetes Care, Mannheim, Germany).

**Figure 2:**
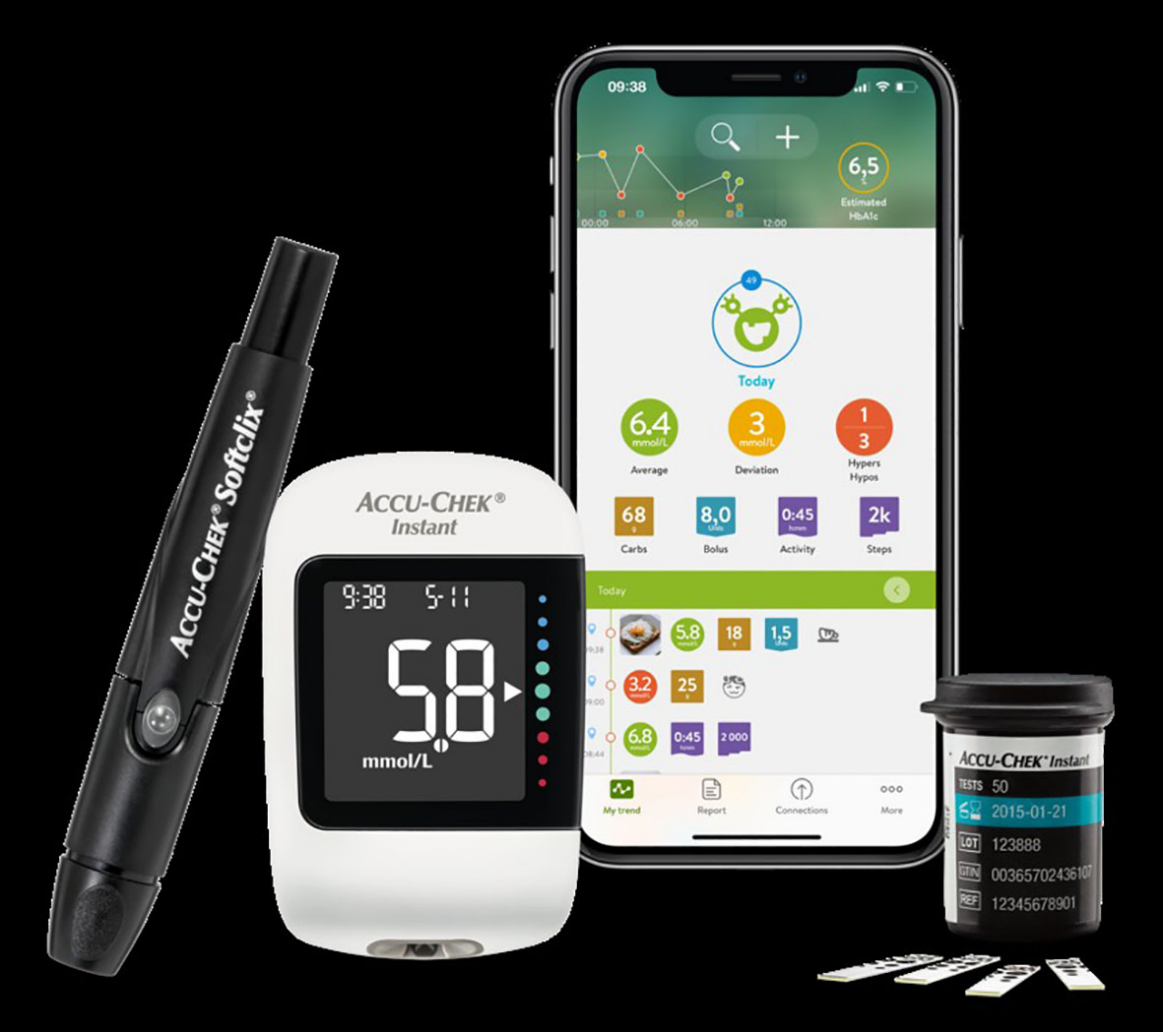
The intervention group used the Accu-Chek^®^ Instant glucometer (Roche Diabetes Care, Mannheim, Germany), which automatically synchronized readings with the MySugr^®^ mobile application (Roche Diabetes Care GmbH, Mannheim, Germany).

The control group used a traditional glucometer (Accu-Chek^®^ Performa glucometer (Roche Diabetes Care, Mannheim, Germany)) (**Figure 1**) with manual recording of blood glucose readings in a physical logbook. The intervention group used a Bluetooth-enabled glucometer (Accu-Chek^®^ Instant glucometer (Roche Diabetes Care, Mannheim, Germany)) that automatically synchronized readings with the MySugr^®^ mobile application (Roche Diabetes Care GmbH, Mannheim, Germany) (**Figure 2**), which they shared with DNEs via WhatsApp Messenger (Meta Platforms, Inc., Version 2.19.10, Menlo Park, CA, USA) or email. All functions of the MySugr^®^ app were accessible to all participants. Refer to **Figure 2** for images of the MySugr^®^ app. During the initial 6-week period, both groups received three biweekly teleconsultations with DNEs, focusing on insulin dose adjustments and individualized diabetes education.

All participants maintained their usual diabetes care throughout the study, including regular clinic visits, blood investigations, diabetes education materials, and hospital support programmes. Capillary blood glucose targets were personalized for each participant based on their age, comorbidities, renal function, and risk of hypoglycemia.

### 2.6 Study outcomes

HbA1c and low-density lipoprotein (LDL) were analyzed at the College of American Pathologists-accredited laboratory (Tan Tock Seng Hospital, Singapore). Cardiometabolic parameters were measured using standardized equipment: blood pressure via the CARESCAPE™ V100 Vital Signs Monitor (GE Healthcare, Chicago, IL, USA), and weight and height using the SECA SCALE UP 360 Ultrasonic Measuring Station with ID-Display and Handrail (SECA GmbH & Co. KG, Hamburg, Germany), from which body mass index (BMI) was calculated.

Patient-reported outcomes were assessed using two validated instruments. The 15-item Glucose Monitoring System Satisfaction (GMSS) scale measured device satisfaction, with higher mean scores (after reverse coding negative items) indicating greater satisfaction. The Problem Areas in Diabetes (PAID) scale evaluated diabetes-related emotional distress, with higher scores indicating increased distress. Phone consultation times, emergency department visits, and adverse glycemic events were also assessed.

### 2.7 Data collection timeline

The study comprised an initial six-week intervention period with fortnightly teleconsultations, followed by an 18-week follow-up period incorporating clinic visits at weeks 12 and 24. Clinical measurements, including HbA1c and cardiometabolic parameters (blood pressure, weight, height, and BMI), were assessed at three time points: baseline, week 12, and week 24. Primary outcome was HbA1c, with the final endpoint at week 24. Low-density lipoprotein (LDL) cholesterol levels, Glucose Monitoring System Satisfaction (GMSS) scores, and Problem Areas in Diabetes (PAID) scores were collected at baseline and week 24.

### 2.8 Statistical analysis

Statistical analyses were conducted on an intention-to-treat basis using IBM SPSS Statistics for Windows, Version 26.0 (IBM Corp., Armonk, NY, USA). In the event of withdrawal or loss to follow-up, patients were still included in the analysis for the duration they were observed. Means with SD were reported for all the numerical variables, while frequency and percentage were reported for all the categorical variables. The Chi-square test was used to compare demographics, socioeconomics, and baseline clinical characteristics. The HbA1c reduction at weeks 12 and 24 from baseline was summarized by mean and SD, and a generalized linear model was used to compare it between BTG and TG to adjust for the baseline HbA1c level. Moreover, a paired t-test was used to compare the HbA1c levels measured at weeks 12 and 24 with baseline levels within each intervention group. The cardiometabolic variables and quality of life outcomes were analyzed similarly. The safety outcomes, including the incidence of adverse events and insulin titration, were compared using the Chi-square test. A two-sided p<0.05 was considered statistically significant. GMSS and PAID outcome endpoints were compared by using 2 sample T test and followed by general linear model to adjust for the baseline measurements. An ANCOVA was conducted to compare changes in PAID and GMSS scores between the intervention and control groups while adjusting for baseline PAID scores.

## 3 Results

**Figure 3:**
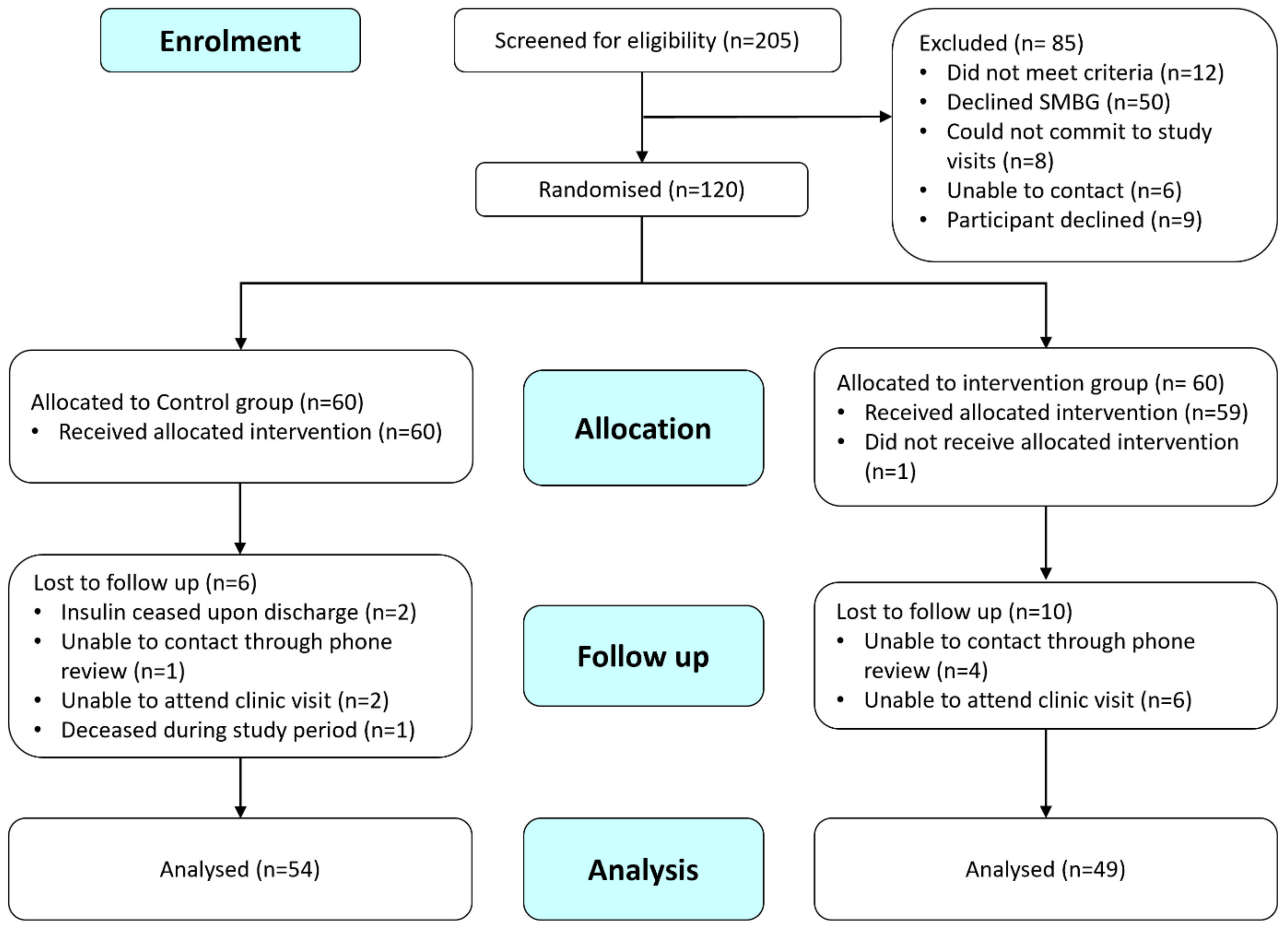
CONSORT Flow Diagram.

One hundred and twenty participants were recruited for this study - 17 participants dropped out, and 103 participants completed the study (**Figure 3**).

### 3.1 Baseline clinical characteristics

**Table 1** presents comparable baseline characteristics across the groups. The study population was predominantly male (68.3%), Chinese (62.5%), and diagnosed with Type 2 diabetes (85.8%). Most participants had a diabetes duration of less than 10 years and were new to insulin therapy. Basal insulin was the most common prescribed regimen, and participants were typically advised to perform SMBG 6 times per week.

**Table 1:** Demographic and baseline characteristics of participants (n=120).

Demographic data	Intervention n (%)	Control n (%)	*P*-value
Age (years)			0.200
21 - 50	31 (51.7)	24 (40.0)	
51 - 70	29 (48.3)	36 (60.0)	
Gender			1.000
Female	19 (31.7)	19 (31.7)	
Male	41 (68.3)	41 (68.3)	
Race			0.412
Chinese	37 (61.7)	38 (63.3)	
Malay	16 (26.7)	10 (16.7)	
Indian	6 (10.0)	11 (18.3)	
Others	1 (1.7)	1 (1.7)	
Living arrangement			0.707
Stays Alone	6 (10.0)	9 (15.0)	
Stays with Friends	4 (6.7)	4 (6.7)	
Stays with Family	50 (83.3)	47 (78.3)	
Employed			0.195
Yes	49 (81.7)	43 (71.7)	
No	11 (18.3)	17 (28.3)	
Carer			0.171
Self	59 (98.3)	56 (93.3)	
Others	1 (1.7)	4 (6.7)	
Type of diabetes			0.191
Type 1	11 (18.3)	6 (10.0)	
Type 2	49 (81.7)	54 (90.0)	
Duration of diabetes			0.426
<10 years	41 (68.3)	42 (70.0)	
10–20 years	11 (18.3)	14 (23.3)	
>20 years	8 (13.4)	4 (6.7)	
Characteristic of insulin therapy			0.409
Newly initiated	41 (68.3)	45 (75.0)	
Intensified	12 (20.0)	12 (20.0)	
Dosage change	7 (11.7)	3 (5.0)	
Insulin regime			0.180
Basal	36 (61.0)	41 (68.3)	
Basal bolus	22 (37.3)	14 (23.3)	
Premixed insulin	4 (6.7)	5 (4.2)	
Basal Plus	0 (0.0)	1 (1.7)	
Prescribed SMBG^1^ per week			0.468
6 SMBG^1^/week	40 (66.7)	42 (70.0)	
9–18 SMBG^1^/week	19 (31.7)	15 (25.0)	
12 SMBG^1^/week	1 (1.7)	3 (5.0)	
Average HbA1c (%)	10.33	11.00	0.031

^1^Self-Monitoring of Blood Glucose.

### 3.2 SMBG adherence

An 80% adherence rate to SMBG is recommended to achieve blood glucose control. Participants initially demonstrated 74.4% (n=117) compliance to this recommendation during the first four weeks, which declined to 67.8% (n=115) by week 6.

### 3.3 Primary outcome

Both groups demonstrated significant reductions in HbA1c throughout the study period. The TG group showed a decrease in HbA1c of 2.8% at week 12 and 3.1% at week 24 (both *p* < 0.001), while the BTG group achieved reductions of 2.23% and 2.18% respectively (both *p* < 0.001) (**Table 2**). After adjusting for baseline HbA1c levels, the general linear model showed that there was no significant difference in HbA1c reduction from baseline between participants in the BTG group and TG group at both week 12 (mean difference: 0.21, 95% CI: -0.28 – 0.71, *p* = 0.402) and week 24 (mean difference: 0.23, 95% CI: -0.37 – 0.84, *p* = 0.440) (**Table 3**); even though patients in the TG group seemed to have slightly greater reduction.

**Table 2:** Change in cardiometabolic parameters from baseline.

	Intervention mean (SD)	Control mean (SD)	Adjusted mean difference (Intervention-Control) groups (95% CI)^3^	*P*-value^3^
HbA1c (%)				
Baseline –Wk^1^ 12 (n=53/55^2^)	-2.23 (2.34)	-2.81 (2.11)	0.21 (-0.29, 0.71)	0.402
Baseline –Wk^1^ 24 (n=48/54^2^)	-2.18 (2.62)	-3.05 (2.68)	0.24 (-0.37, 0.85)	0.440
Body weight (kg)				
Baseline –Wk^1^ 12 (n=54/57^2^)	0.60 (4.13)	0.65 (6.56)	0.09 (-1.99, 2.17)	0.933
Baseline –Wk^1^ 24 (n=48/54^2^)	1.67 (4.66)	1.63 (5.48)	-0.46 (-2.48, 1.57)	0.656
BMI^4^ (kg/m^2^)				
Baseline –Wk^1^ 12 (n=54/57^2^)	0.17 (2.66)	-0.10 (2.83)	-0.15 (-1.12, 0.82)	0.762
Baseline –Wk^1^ 24 (n=48/54^2^)	0.64 (2.33)	0.38 (2.11)	-0.19 (-1.09, 0.71)	0.681
SBP^5^ (mmHg)				
Baseline –Wk^1^ 12 (n=53/56^2^)	5.23 (19.86)	0.73 (20.01)	-2.22 (-8.68, 4.24)	0.497
Baseline –Wk^1^ 24 (n=48/53^2^)	4.79 (21.07)	1.13 (25.66)	-1.33 (-8.51, 5.86)	0.715
DBP^6^ (mmHg)				
Baseline –Wk^1^ 12 (n=53/56^2^)	1.02 (11.13)	1.25 (10.36)	1.19 (-2.43, 4.80)	0.517
Baseline –Wk^1^ 24 (n=48/53^2^)	-0.19 (10.55)	-1.00 (11.51)	-0.18 (-3.97, 3.60)	0.924
LDL^7^ (mmol/L)				
Baseline –Wk^1^ 24 (n=45/51^2^)	-0.25 (0.84)	-0.11 (1.12)	0.26 (-0.07, 0.59)	0.123

^1^Week.

^2^Valid cases in intervention group and control group respectively.

^3^General linear model was applied to adjust for baseline measurement.

^4^Body Mass Index.

^5^Systolic Blood Pressure.

^6^Diastolic Blood Pressure.

^7^Low Density Lipoprotein.

**Table 3:** Comparison of phone consultation outcomes between two groups.

		Intervention n (%)	Control n (%)	*P*-value
Insulin titration required^1^				
PC^3^ 1	Yes	46 (78.0)	33 (56.9)	0.015
	No	13 (22.0)	25 (43.1)	
PC^3^ 2	Yes	45 (78.9)	43 (76.8)	0.782
	No	12 (21.1)	13 (23.2)	
PC^3^ 3	Yes	50 (86.2)	45 (78.9)	0.304
	No	8 (13.8)	12 (21.1)	
80% of SMBG reading within target range^1^				
PC^3^ 1	Yes	29 (51.8)	25 (43.9)	0.399
	No	27 (48.2)	32 (56.1)	
	Unknown	0 (0.0)	0 (0.0)	
PC^3^ 2	Yes	28 (48.3)	34 (59.6)	0.467
	No	27 (46.6)	21 (36.8)	
	Unknown	3 (5.2)	2 (3.5)	
PC^3^ 3	Yes	35 (60.3)	35 (61.4)	0.887
	No	20 (34.5)	18 (31.6)	
	Unknown	3 (5.2)	4 (7.0)	
Duration of call for PC^3^ (mins)^2^				
PC^3^ 1		10.0 (5.0, 40.0)	10.5 (2.0, 30.0)	0.493
PC^3^ 2		10.0 (2.0, 57.0)	10.0 (2.0, 35.0)	0.029
PC^3^ 3		9.0 (3.0, 28.0)	10.0 (1.0, 57.0)	0.142

^1^n (%).

^2^median and range.

^3^Phone Consultation.

### 3.4 Cardiometabolic outcomes

Statistically significant within group LDL decreases were seen in the BTG group (mean difference = 0.25 mmol/L, 95% CI: -0.51 to 0.00, p = 0.050), however there was no statistically significant difference in LDL readings between the BTG group and TG group, even after correcting for baseline readings (mean difference = 0.26 mmol/L, 95% CI: -0.07 to 0.59, p = 0.123). Statistically significant within group weight gain were seen in both the BTG group (mean difference = 1.67kg, 95% CI: 0.32 to 3.03, p = 0.017) and TG group (mean difference = 1.63kg, 95% CI: 0.14 to 3.13, p = 0.033). However, the between group differences in weight were statistically insignificant even after adjusting for baseline differences (p = 0.656). Blood pressure remained stable in both groups, with no significant between-group differences. After adjusting for baseline differences, analysis of cardiometabolic parameters revealed no statistically significant differences between groups (**Table 2**).

### 3.5 Phone consultation outcomes

**Table 3** presents the outcomes of phone consultations between groups across three time points (PC 1 to PC 3). Regarding insulin titration requirements, the intervention group showed a significantly higher proportion of participants needing adjustment at PC1 compared to the control group (78% vs 56.9%, *p* = 0.015). However, the TG group required more frequent prandial insulin titration compared to the BTG group (81.0% vs. 47.8%, *p* = 0.023). Although the TG group also showed a higher proportion of basal insulin titration (67.9% vs. 50.0%), this difference was not statistically significant (*p* = 0.064). The proportion of participants achieving ≥80% SMBG readings within the target range showed no significant differences between groups across all time points.

### 3.6 Patient-reported outcomes

An ANCOVA was conducted to compare changes in PAID scores between the intervention and control groups while adjusting for baseline PAID scores. The analysis showed no statistically significant difference in PAID score change between the Intervention group and Control group (95% CI: -4.78, 7.39, *p* = 0.671).

In contrast, baseline PAID scores were a significant predictor of change. For every one-unit increase in baseline PAID score, the change in PAID decreased by 0.44 points (95% CI: -0.63, -0.25, *p* < 0.001), indicating that participants with higher initial emotional distress experienced greater reductions over time.

To assess the effect of the intervention on the change in GMSS scores, adjusting for baseline GMSS scores. There was no statistically significant difference in GMSS score change between the Intervention group and the Control group (95% CI: -4.61, 1.62, *p* = 0.345), indicating a negligible effect of the intervention.

However, the baseline GMSS score was a significant covariate. For every one-unit increase in baseline GMSS, the change in GMSS decreased by 0.684 points (95% CI: -0.90 to -0.46, *p* < 0.001). This suggests that participants with higher baseline satisfaction reported smaller increases (or larger decreases) in satisfaction over time.

### 3.7 Adverse events

Adverse events were generally less frequent in the BTG group. Although the TG group showed higher rates of hyperglycemia (25.9% vs. 16.3%, *p* = 0.235) and hypoglycemia (18.5% vs. 12.2%, *p* = 0.380), these differences were not statistically significant. Notably, emergency department visits were significantly lower in the BTG group compared to the TG group (4.1% vs 16.7%, *p* = 0.039). Hospital admissions, although more frequent in the TG group (21.8% vs 12.2%), did not reach statistical significance (*p* = 0.198). Detailed results are presented in **Table 4**.

**Table 4:** Comparison of adverse events among two groups.

Adverse events	Intervention n (%)	Control n (%)	*P*-value
Hypoglycemia^1^			
Week 12	4 (7.4)	6 (10.5)	0.566
Week 24	3 (6.1)	7 (13.0)	0.242
Overall	6 (12.2)	10 (18.5)	0.380
Hyperglycemia^1^			
Week 12	4 (7.4)	10 (17.9)	0.100
Week 24	6 (12.2)	6 (11.1)	0.858
Overall	8 (16.3)	14 (25.9)	0.235
ED^2^ visit^1^			
Week 12	2 (3.7)	4 (7.0)	0.440
Week 24	0 (0.0)	5 (9.3)	0.029
Overall	2 (4.1)	9 (16.7)	0.039
Hospital admission^1^			
Week 12	2 (3.7)	7 (12.3)	0.098
Week 24	4 (8.2)	7 (13.0)	0.431
Overall	6 (12.2)	12 (21.8)	0.198

^1^n (%).

^2^Emergency Department.

## 4 Discussion

The implementation of BTG in diabetes management presents a nuanced landscape of benefits and limitations. While this study showed that BTG did not demonstrate superior glycemic control compared to traditional methods, it does support previous research that self-monitoring of blood glucose with telehealth, regardless of the use of Bluetooth, facilitates better glycemic control (15, 16) and improved LDLs (17) Most notably, the results highlight the importance of telehealth and also indicate significant advantages of BTG in optimising insulin titration, healthcare resource utilisation and safety, which are key areas of effective preventive telehealth monitoring.

### 4.1 The importance of telehealth in HbA1c

The results showed that when it comes to glycemic control, especially in terms of HbA1c, the role of effective telehealth monitoring may be more important than using BTG. Previous research has suggested significant advantages of connected glucose monitoring devices, with Grady et al. (16) reporting improved glucose readings in range by 8.1-11.2% amongst over 17, 000 people with diabetes, and Xiao et al. (18) demonstrating effective improvements in blood glucose levels and BMI through digital diabetes management. However, this study found no statistically significant glycaemic superiority of BTG over traditional glucometers within a structured telehealth framework. Instead, this finding corroborates with established evidence that SMBG facilitates HbA1c reduction (15); however, it suggests that the benefit of Bluetooth connectivity may be modest at best when both groups receive structured telehealth insulin titration support. The results indicate that within a robust telehealth framework, the primary contributor to improved glycemic outcomes may be the frequency and timeliness of professional feedback rather than the device’s connectivity feature alone. This observation aligns with findings from Xiao et al. (18), which demonstrated that digital diabetes management effectively improves blood glucose levels in individuals with T2DM in home settings, with success primarily attributed to frequent SMBG supported by dedicated healthcare professionals providing timely, personalised, and responsive guidance.

### 4.2 Better optimised insulin titration

A notable finding was that the BTG group required more insulin titration adjustments initially, compared to the TG group. A previous study suggested that early insulin titration, especially within a 12-week period, can lead to better glycemic control as it achieves glycemic targets faster and help preserve beta-cell function (19) This may reflect the advantages of real-time glucose data transmission. Immediate access to comprehensive glucose profiles likely enabled DNEs to detect patterns earlier and initiate titration decisions more confidently. Conversely, the TG group relied on manual transcription of glucose readings, a process prone to incomplete data or inaccuracies. Over time, this study’s results also showed that the TG group required more prandial insulin titration than the BTG group. This might be due to better awareness of the BTG participants’ glycemic control as the app also shows colour-coded CBG targets (20) These findings suggest that BTG optimises insulin titration processes and reinforce that structured professional support remains the cornerstone of effective diabetes management regardless of monitoring technology employed.

### 4.3 Improved healthcare utilization and safety

A particularly significant outcome was the marked reduction in emergency department (ED) utilisation among BTG participants, attributed to more frequent and timely insulin modifications during telehealth consultations. Automated glucose data transmission enabled continuous monitoring by DNEs and proactive dosage adjustments, thereby minimising both hyperglycaemic and hypoglycaemic events. The BTG system’s comprehensive interface of incorporating colour-coded glycaemic targets, estimated HbA1c projections, trend visualisation and integrated bolus calculators likely also empowered participants to interpret their data and engage proactively in self-management. This automatic data transmission addresses critical safety concerns, as Geller et al. (21) identified insulin-related errors as major contributors to ED presentations through inappropriate dosing or administration. Considering that ED visits generate approximately $1387 in patient costs per episode in the United States (22) while exacerbating strain on overburdened emergency services (23), these reductions in healthcare utilisation have substantial implications for resource optimisation and cost-effectiveness across healthcare systems and patient populations. These outcomes are consistent with extensive real-world data demonstrating that enhanced data connectivity and professional feedback loops constitute primary mechanisms through which connected glucose monitoring technologies improve clinical outcomes (21, 24).

### 4.4 Weight gain: a side-effect of better glycemic control

Both the BTG and TG groups experienced statistically significant weight gain, with a slightly greater (though non-significant) increase in the BTG arm. This finding contrasts with evidence from Xiao et al. (18), which demonstrated that digital diabetes management can effectively improve both blood glucose levels and BMI in individuals with T2DM. The observed weight gain in this study where both groups received insulin titration is likely due to a combination of the anabolic effect insulin has in inhibiting protein breakdown and increased calorie intake by participants trying to prevent hypoglycemia (23). The results may therefore reflect intensified insulin therapy or lifestyle factors associated with tighter glucose regulation rather than device-related effects. Further investigation is warranted to determine how digital tools influence weight trajectories within intensive telehealth support.

## 5 Conclusion

In summary, the IT-PDM study demonstrates that within a structured telehealth framework, BTG enhances therapeutic responsiveness, healthcare utilization and patient safety. Moreover, the addition of Bluetooth connectivity via an app did not negatively impact glucose device satisfaction, contributing to the feasibility to the implementation of BTG. However, Bluetooth-enabled glucometers did not confer significant glycemic superiority over traditional monitoring. These findings align with broader evidence that the success of digital diabetes management depends primarily on timely clinician engagement, real-time data utilization and patient support rather than the connectivity feature alone. Bluetooth connectivity thus serves as an enabler of coordinated, responsive and safer telehealth-based diabetes care.

### 5.1 Limitations and future directions

Several limitations should be considered when interpreting our findings. As a single-centre study conducted at a tertiary hospital in Singapore, the results may not be fully generalisable to other healthcare settings or populations. The open-label design, necessitated by the nature of the intervention, could have introduced bias in self-reported outcomes. Our requirement for smartphone ownership and familiarity with mobile applications may have selected for a more tech-savvy population, potentially overestimating the feasibility of BTG implementation in the broader diabetes population. The 24-week follow-up period, while sufficient to demonstrate glycemic improvements, may not fully capture long-term adherence and sustainability of the intervention. The predominance of male, Chinese participants with relatively short diabetes duration may restrict generalizability to other demographic groups or those with longer-standing diabetes. Finally, the study did not systematically capture technical challenges or user difficulties with the BTG system, which could affect real-world implementation.

Future research should examine specific patient subgroups, particularly elderly patients and those with limited technological literacy, to identify populations that would benefit most from BTG technology. Additionally, studies should explore how these systems can be optimally integrated into existing healthcare workflows to reduce healthcare utilisation and enhance clinical efficiency.

## Data Availability

The original contributions presented in the study are included in the article/**Supplementary Material**. Further inquiries can be directed to the corresponding author.
