# Supplementary material for: Telehealth insulin titration in adults with diabetes: a randomized controlled trial comparing bluetooth-enabled versus traditional glucometers
Source: Front Endocrinol (Lausanne). 2025 Dec 16;16:1724811. doi: 10.3389/fendo.2025.1724811 (PMC12747901; doi:10.3389/fendo.2025.1724811)
Supplement: Supplementary file 1 [file DataSheet1.docx]

**Supplementary Materials**

| **Supplementary Data Sheet 1** | Guideline for CBG Monitoring |
| --- | --- |
| **Supplementary Data Sheet 2** | Guideline for Insulin Titration |
| **Supplementary Data Sheet 3** | Table Comparing Cardiometabolic Outcomes at Various Study Time Points |

**Supplementary Data Sheet 1**

**Guideline for CBG Monitoring**

Step 1: Ascertain HbA1c Target

HbA1c targets should be individualized and specified by the primary team doctor

Consider the following when determining glycemic targets:

Co-existing chronic illnesses e.g. Organ failure, cancer

Risk of hypoglycemia/ hypoglycemia unawareness

Frailty

Limited life expectancy

Step 2: Frequency of CBG Monitoring

The healthcare providers (HCPs) should have advised the patient on the frequency of CBG monitoring

|  | SMBG check timings | Frequency | Minimum no. of checks/week |
| --- | --- | --- | --- |
| **Basal insulin only** | Pre-breakfast,  pre-dinner | At least on  3 consecutive days of the week | 6 |
| **Basal**  **+ Prandial insulin** | As per basal,  plus 2-hours post meals |  | 9 to 18 |
| **Pre-mixed insulin** | Pre and post meals |  | 12 |

Factors to consider include:

- Type of insulin regime
- Underlying DM pathology
- Risk of hypoglycemia
- Prandial hyperglycemia
- Logistic reasons

Step 3 Fasting and Prandial CBG Targets

| **HbA1c Targets** | **Fasting/ Pre-meal Targets** | **2-hour Prandial Targets** |
| --- | --- | --- |
| 6.5 - 7.5% | 4.0 – 8.0 mmol/L | 7.0 – 9.0 mmol/L |
| 7.5 – 8.5% | 8.1 – 10.0 mmol/L | 9.1 – 12.0 mmol/L |
| > 8.5% | 10.1 – 12.0 mmol/L | 12.1 – 15.0 mmol/L |

**Supplementary Data Sheet 2**

**Guideline for Insulin Titration**

DNEs can titrate insulin using the CBG targets depending on the pre-set HbA1c targets.

| **HbA1c Targets** | **Fasting/ Pre-meal Targets** | **2-hour Prandial Targets** |
| --- | --- | --- |
| 6.5 - 7.5% | 4.0 – 8.0 mmol/L | 7.0 – 10.0 mmol/L |
| 7.5 – 8.5% | 8.1 – 10.0 mmol/L | 10.1 – 13.0 mmol/L |
| > 8.5% | 10.1 – 12.0 mmol/L | 13.1 – 15.0 mmol/L |

| **Insulin Titration** | |
| --- | --- |
| **If CBG is at target** | Maintain dose |
| **If CBG is below target/ hypoglycemia** | Evaluate for possible causes of hypoglycemia.  Consider insulin dose reduction.   - If fasting CBG is low: reduce night basal/ predinner insulin - If premeal CBG is low: reduce preceding meal basal/prandial insulin - If prandial CBG is low: reduce current meal prandial insulin - If patient is on concurrent sulphonylureas, consider dose reduction as well.   Amount of insulin to reduce   - Patients with high risk of hypoglycemia: reduce dose by 20% - Patients with low risk of hypoglycemia: reduce dose by 10% |
| **If CBG is above target** | Evaluate for possible causes of hyperglycemia.  Observe for a CBG trend for 3-5 days.  Consider insulin dose increment for persistently high CBG.  Do not increase insulin based on a single reading.   - If fasting CBG is high: increase night basal/ predinner insulin - If premeal CBG is high: increase preceding meal basal/prandial insulin - If prandial CBG is high: increase current meal prandial insulin - If CBG is above target for most premeal readings: increase basal insulin   Amount of insulin to increase   - Patients with high risk of hypoglycemia: increase dose by 10% - Patients with low risk of hypoglycemia: increase dose by 20%   If dose of basal insulin is > 50% of body weight, refer to doctor to consider starting prandial insulin or other adjunctive therapy. |

**In events of severe hyperglycemia (CBG > 25mmol/L), ask for signs and symptoms of DKA/ HHS.

-If present, patients must be advised to come to the emergency department immediately.

-If absent, inform the doctor and consider reviewing the patient in the clinic at the earliest date.

**Supplementary Data Sheet 3**

Table Comparing Cardiometabolic Outcomes at Various Study Time Points^1^

|  | Intervention | | | Control | | | |  |
| --- | --- | --- | --- | --- | --- | --- | --- | --- |
|  | **Mean±SD** | **95% CI** | **p-value** | | **Mean±SD** | **95% CI** | **p-value** | |
| **HbA_1c_ (%)** |  |  |  | |  |  |  | |
| Wk^2^ 12-Baseline | -2.23±2.34 | -2.87, -1.59 | <0.001 | | -2.81±2.11 | -3.38, -2.24 | <0.001 | |
| Wk 24-Baseline | -2.18±2.62 | -2.94, -1.42 | <0.001 | | -3.05±2.68 | -3.78, -2.32 | <0.001 | |
| Wk 24 -Wk 12 | -0.18±0.97 | -0.45, 0.10 | 0.212 | | -0.26±1.19 | -0.58, 0.70 | 0.119 | |
| **Body weight (kg)** |  |  |  | |  |  |  | |
| Wk 12-Baseline | 0.60±4.13 | -0.52, 1.73 | 0.287 | | 0.65±6.56 | -1.10, 2.39 | 0.461 | |
| Wk 24-Baseline | 1.67±4.66 | 0.32, 3.03 | 0.017 | | 1.63±5.48 | 0.14, 3.13 | 0.033 | |
| Wk 24 -Wk 12 | 0.79±2.93 | -0.06, 1.64 | 0.069 | | 1.05±5.71 | -0.51, 2.61 | 0.182 | |
| **BMI^3^ (kg/m^2^)** |  |  |  | |  |  |  | |
| Wk 12-Baseline | 0.17±2.66 | -0.56, 0.89 | 0.649 | | -0.10±2.83 | -0.85, 0.66 | 0.798 | |
| Wk 24-Baseline | 0.64±2.33 | -0.04, 1.32 | 0.064 | | 0.38±2.11 | -0.20, 0.95 | 0.196 | |
| Wk 24 -Wk 12 | 0.14±1.13 | -0.19, 0.47 | 0.389 | | 0.48±2.55 | -0.22, 1.17 | 0.176 | |
| **SBP^4^ (mmHg)** |  |  |  | |  |  |  | |
| Wk 12-Baseline | 5.23±19.86 | -0.25, 10.70 | 0.061 | | 0.73±20.01 | -4.63, 6.09 | 0.785 | |
| Wk 24-Baseline | 4.79±21.07 | -1.33, 10.91 | 0.122 | | 1.13±25.66 | -5.94, 8.20 | 0.749 | |
| Wk 24 -Wk 12 | 1.72±15.27 | -2.76, 6.21 | 0.443 | | 0.71±24.16 | -6.02, 7.44 | 0.833 | |
| **DBP^5^ (mmHg)** |  |  |  | |  |  |  | |
| Wk 12-Baseline | 1.02±11.13 | -2.05, 4.09 | 0.508 | | 1.25±10.36 | -1.52, 4.02 | 0.370 | |
| Wk 24-Baseline | -0.19±10.55 | -3.25, 2.88 | 0.903 | | -1.00±11.51 | -4.17, 2.17 | 0.530 | |
| Wk 24 -Wk 12 | -0.55±8.02 | -2.91, 1.80 | 0.639 | | -1.82±11.20 | -4.94, 1.29 | 0.245 | |

| **LDL^6^ (mmol/L)** |  |  |  |  |  |  | |
| --- | --- | --- | --- | --- | --- | --- | --- |
| Wk 24-Baseline | -0.25±0.84 | -0.51, 0.00 | 0.050 | -0.11±1.12 | -0.43, 0.20 | 0.476 |  |

*^1^ Paired T-test was conducted*

*^2^ Week*

*^3^ Body Mass Index*

*^4^ Systolic Blood Pressure*

*^5^ Diastolic Blood Pressure*

*^6^ Low Density Lipoprotein*
